# Chloroquine has shown high therapeutic efficacy against uncomplicated *Plasmodium vivax* malaria in southern Ethiopia: seven decades after its introduction

**DOI:** 10.1186/s12936-024-05009-7

**Published:** 2024-06-10

**Authors:** Anteneh Kassahun Mare, Hussein Mohammed, Heven Sime, Henok Hailgiorgis, Kale Gubae, Bekuretsion Gidey, Mebrahtom Haile, Gudissa Assefa, Worku Bekele, Sarah Auburn, Rick Price, Jonathan B. Parr, Jonathan J. Juliano, Geremew Tasew, Solomon Mequanente Abay, Ashenafi Assefa

**Affiliations:** 1 Department of Pharmacology and Clinical Pharmacy, School of Pharmacy, College of Health Sciences, Addis Ababa University, P.O. Box 9086, Addis Ababa, Ethiopia; 2 Malaria and Other Parasitic Diseases Research Team, Bacterial, Parasitic and Zoonotic Diseases Research Directorate, Ethiopian Public Health Institute, Addis Ababa, Ethiopia; 3 Department of Pharmacy, College of Health Sciences, Debre Markos University, Debre Markos, Ethiopia; 4 Ethiopian Ministry of Health, Addis Ababa, Ethiopia; 5 World Health Organization, Addis Ababa, Ethiopia; 6 Global and Tropical Health Division, Menzies School of Health Research and Charles Darwin University, Darwin, NT Australia; 7 Institute of Infectious Disease and Global Health, University of North Carolina at Chapel Hill, Chapel Hill, USA

**Keywords:** *Plasmodium vivax*, Chloroquine, Therapeutic efficacy, Arba Minch, Ethiopia

## Abstract

**Background:**

*Plasmodium vivax* malaria is a leading cause of morbidity in Ethiopia. The first-line treatment for *P. vivax* is chloroquine (CQ) and primaquine (PQ), but there have been local reports of CQ resistance. A clinical study was conducted to determine the efficacy of CQ for the treatment of *P. vivax* malaria in southern Ethiopia.

**Methods:**

In 2021, patients with *P. vivax* mono-infection and uncomplicated malaria were enrolled and treated with 25 mg/kg CQ for 3 consecutive days. Patients were followed for 28 days according to WHO guidelines. The data were analysed using per-protocol (PP) and Kaplan‒Meier (K‒M) analyses to estimate the risk of recurrent *P. vivax* parasitaemia on day 28.

**Results:**

A total of 88 patients were enrolled, 78 (88.6%) of whom completed the 28 days of follow-up. Overall, 76 (97.4%) patients had adequate clinical and parasitological responses, and two patients had late parasitological failures. The initial therapeutic response was rapid, with 100% clearance of asexual parasitaemia within 48 h.

**Conclusion:**

Despite previous reports of declining chloroquine efficacy against *P. vivax*, CQ retains high therapeutic efficacy in southern Ethiopia, supporting the current national treatment guidelines. Ongoing clinical monitoring of CQ efficacy supported by advanced molecular methods is warranted to inform national surveillance and ensure optimal treatment guidelines.

## Background

According to the World Health Organization (WHO), in 2021, there were approximately 247 million malaria cases in 84 malaria-endemic countries. Approximately 95% of cases and 96% of deaths occur in African countries globally [1]. In Africa, the predominant burden of malaria is attributable to *Plasmodium falciparum*; however, *Plasmodium vivax* is endemic in several areas, including the Horn of Africa and the Sahel region [2].

In Ethiopia, almost 40% of malarial cases are caused by *P. vivax* [3, 4]. Malaria transmission occurs mainly at altitudes less than 2000 m [5, 6]. Malaria transmission is seasonal, and in most parts of the country, the main transmission season is from September to December, following the main rainy season from June to August. A second minor transmission season occurs during April and May and is usually limited to the Eastern and Southeastern regions, which experience short rains from February to March [7]. The first-line treatment for *P. vivax* malaria in Ethiopia is chloroquine (CQ) and primaquine (PQ); PQ is used for radical curing by clearing the hypnozoite stage from the liver [7]. In the current study we reported the therapeutic efficacy of CQ only. We have provided 14 days PQ as per the guideline.

Anti-malarial drug resistance is a global threat to the effective control of malaria. Hence, robust anti-malarial surveillance is necessary to determine the therapeutic efficacy of current treatment regimens so that policy makers can optimize treatment guidelines. Surveillance of chloroquine-resistant parasites is largely informed by clinical efficacy studies, but these studies are confounded by an inability to reliably distinguish between recrudescent infections, relapses and reinfections [8]. In contrast to *P. falciparum*, there are no validated molecular markers of CQ resistance (CQR) in *P. vivax,* and ex vivo drug susceptibility assays are logistically challenging [8]. Although CQR has been found in many endemic areas, the degree to which treatment failure is attributable to drug resistance, poor absorption or underdosing of chloroquine is unclear [9, 10]. CQ remains the first-line drug for *P. vivax* infection in most *P. vivax* endemic countries, including Ethiopia. However, in Malaysia, Indonesia and Papua New Guinea, where the risk of recurrence following CQ exceeds 60%, control programmes have changed the first-line policy for patients presenting with vivax malaria to artemisinin-based combination therapy (ACT) [11–16]. Some clinical studies have evaluated the therapeutic efficacy of CQ for the treatment of *P. vivax* in Ethiopia [17–25]. A study reported a 21.9% treatment failure at one of its four sentinel sites [19], another study reported 13% treatment failure rate [20] whereas others reported that the efficacy of these treatments exceeded 95% [17, 18, 21–25]. Thus, the true burden of CQR *P. vivax* in Ethiopia remains unclear. To provide additional data about the efficacy of CQ in Ethiopia, a therapeutic efficacy survey (TES) of CQ was conducted in Arba Minch, southern Ethiopia.

## Methods

### Study design

This study was a one-arm prospective evaluation of clinical and parasitological responses to directly observed treatment with CQ for uncomplicated *P. vivax* malaria.

### Study area and period

**Fig. 1:**
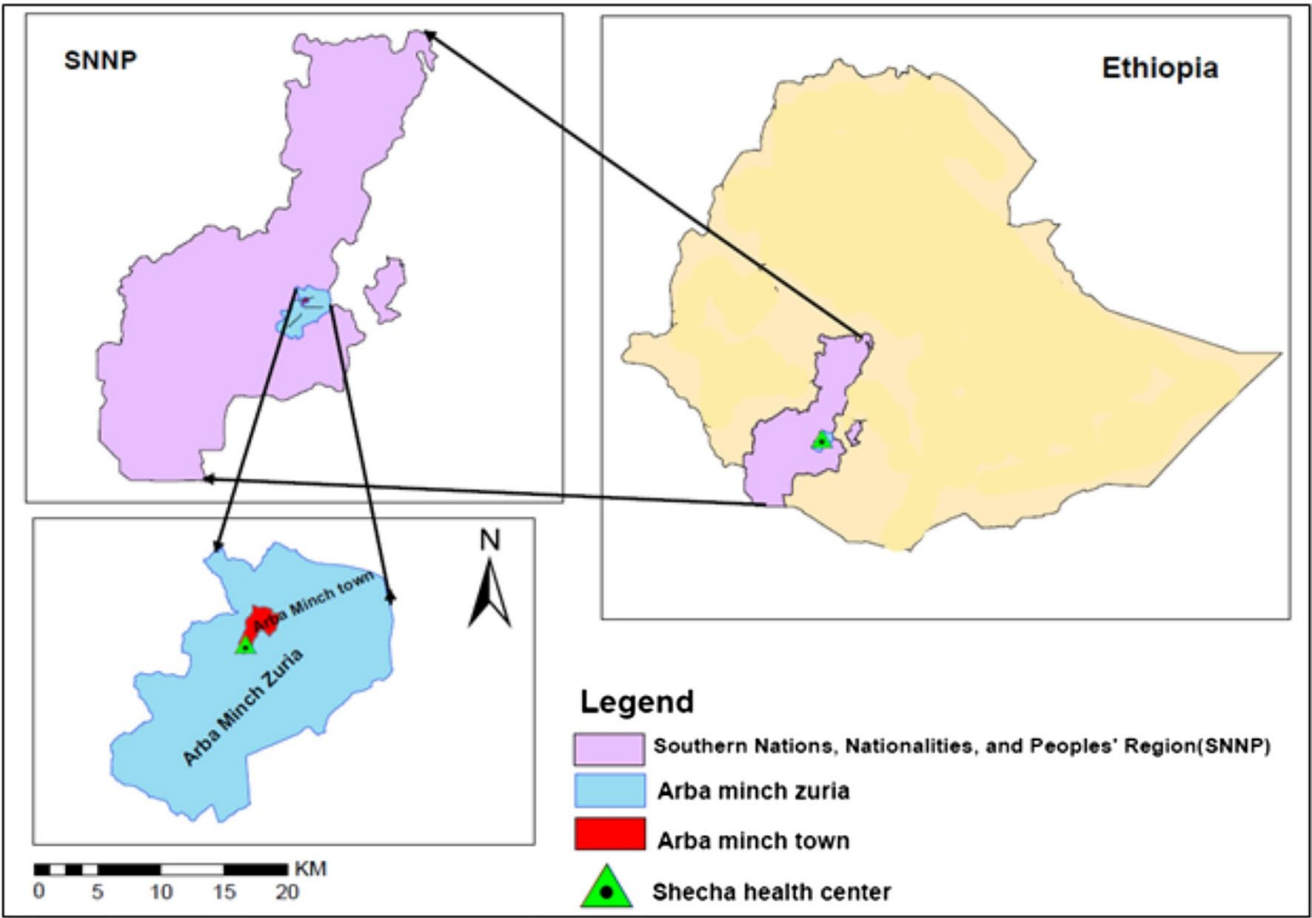
Map of the study area

The study was carried out at the Shecha Health Centre in Arba Minch town, southern Ethiopia, between March and August 2021 (Fig. 1). Arba Minch is administratively located in the Gamo Gofa zone of the Southern Nations, Nationalities, and Peoples Region, approximately 500 km south of Addis Ababa, Ethiopia's capital city. Arba Minch is located at 6.03333 latitude and 37.55 longitude. The town is at an elevation of 1200–1300 m above sea level, and its annual temperature is 29.7 °C, with an average annual rainfall of 800–1000 mm. Malaria is a common cause of morbidity and mortality. The prevalence of malaria in a survey conducted in 2010 was 7%, with 64% of malaria due to *P. falciparum,* 25% due to *P. vivax* and 11% due to mixed infections [26]*. Anopheles arabiensis* is the main malaria vector in Ethiopia [27].

### Study population

Patients aged older than 6 months and presenting with uncomplicated malaria due to *P. vivax* mono-infection at the Shecha Health Centre were eligible for inclusion in the study.

### Sample size

The sample size was calculated based on a previous study conducted in the area (Shele, Southern Ethiopia), which reported 3.8% treatment failure [19] with 5% precision and a 95% confidence level. A calculated sample size of 56 was determined based on N = (Z/d)2 P (1-P) [28]. An additional 20% enrolment was added for the expected withdrawal and loss to follow-up cases during the full 28-day study period. A total of 88 subjects were included in this study.

### Inclusion/exclusion criteria

The inclusion criteria included age > 6 months, mono-infection with *P. vivax* detected by microscopy, asexual parasite count > 250/μl, axillary temperature ≥ 37.5 °C or history of fever during the 48 h before recruitment, ability to swallow oral medication, ability and willingness to comply with the study protocol for the duration of the study and to comply with the study visit schedule. All patients provided informed consent or assent from the patient and/or from a parent or legal guardian for individuals < 18 years of age.

The exclusion criteria included the presence of danger signs [29] in children aged less than 5 years or with signs of severe malaria or mixed or monoinfected with another *Plasmodium* species. Pregnant women or those who were breastfeeding were excluded. Patients with febrile illness due to diseases other than malaria or other known underlying chronic or severe diseases (e.g., cardiac, renal and hepatic diseases); those taking medications known to interfere with the pharmacokinetics of chloroquine; and those with a history of hypersensitivity reactions or contraindications to chloroquine were also excluded.

### Treatment and follow-up

A 28-day CQ efficacy study was conducted according to WHO guidelines [29]. CQ (Lariago DS®, batch number 80368, Remedica Ltd-Cyprus) was administered at a total dose of 25 mg base/kg for three consecutive days (10 mg/kg on days 0 and 1 and 5 mg/kg on day 2), and the parasitological and/or clinical response was monitored for 28 days. CQ was administered as a direct observation therapy. Clinical assessments, including adverse events, were recorded at each visit. After CQ administration, patients were observed for 30 min, and those who vomited within 30 min were retreated with the same dose. Patients who vomited twice were excluded from the study. Patients were followed up on days 1, 2, 3, 7, 14, 21, and 28. At each visit except for day 1, a blood smear was taken for microscopic examination. The haemoglobin (Hb) concentration was measured at baseline and again on days 14 and 28. The patients haematocrit data was converted to Hb using the formula: Hb (g/dL) = (haematocrit [%] – 5.62)/2.60 [30].

### Parasite identification

Peripheral parasitaemia was detected by microscopic examination of thick and thin blood smears. The initial staining was performed quickly (10% Giemsa, 15 min). Following the patient's enrolment, the second thick blood smear was stained slowly (3% Giemsa for 45 min). A hand tally counter and a predetermined number of white blood cells (typically 200) were used to determine the parasite density. The parasite counts on consecutive blood films (on days 2, 3, 7, 14, 21, and 28) were determined using the same method. When examination of 1000 white blood cells revealed no asexual parasites, blood film examination was recorded as negative. The number of asexual parasites per microlitre of blood was determined by dividing the number of asexual parasites by the number of white blood cells counted and multiplying by an assumed white blood cell density (8,000 per microlitre). Two qualified microscopists read all the slides independently, and parasite densities were calculated by averaging the two counts.

### Quality assurance

All slides were re-examined by two expert WHO-certified microscopists for quality control, and during the analysis (differences between the two microscopists with a parasite density > 50%), a third expert microscopist re-examined the slides, and parasite density was calculated by averaging the two closest counts.

### Withdrawal

To classify patients as withdrawn, the following general criteria were established: withdrawal of consent; failure to complete treatment (due to persistent vomiting during the treatment, failure to attend scheduled visits during the first three days, or serious adverse events necessitating treatment termination before the full course is completed); severe malaria on day 0; inclusion of a patient who does not meet the inclusion criteria; self- or third-party administration of drugs with anti-malarial activity; detection of a mono-infection with another malaria species during follow-up; or misclassification of a patient due to a laboratory error (parasitaemia).

### Clinical endpoints

Efficacy endpoints were classified based on the parasitological and clinical outcomes of anti-malarial treatment according to the World Health Organization (WHO) guidelines [29].

The nature and frequency of adverse events were recorded to assess safety. Every patient was routinely questioned about past symptoms as well as any new symptoms that had developed since their last visit. Fever was defined as an axillary temperature ≥ 37.5 °C.

### Statistical analysis

The data were entered into the WHO-designed Excel spreadsheet and analysed using SPSS version 26 for Windows (Chicago, USA). To determine the efficacy of CQ, data were analysed using two methods: per-protocol (PP) analysis and the Kaplan‒Meier (K‒M) method. The PP analysis included all enrolled patients, excluding patients who did not meet the enrolment criteria; who failed to complete treatment (due to persistent vomiting, failure to attend scheduled visits during the first 3 days, or serious adverse events necessitating termination of treatment); and who did not complete follow-up either because they were lost to follow-up or had recurrent parasitaemia due to mono-infection with another parasite species.

Patients were also withdrawn from the analysis if they had severe malaria at baseline, did not meet the inclusion criteria, had self- or third-party administration of drugs with anti-malarial activity, had mono-infection with another malaria species during follow-up, or had a misclassification of a patient due to a laboratory error (parasitaemia).

Loss to follow-up occurred when, despite all reasonable efforts, an enrolled patient did not attend scheduled visits and could not be located or when the protocol was violated for more than one day. These patients experienced no treatment outcome. These patients were considered lost to follow-up and were either censored or excluded from the analysis.

K‒M survival analysis was used to calculate the cumulative risk of recurrence on day 28 in patients who met all of the enrolment criteria or completed treatment. Patients were included until they were censored on their last day of follow-up, when they were lost to follow-up, had recurrence with mono-infection of another infection, or reached the end of the follow-up.

Parasite and fever clearance rates, mean geometric parasite density and mean Hb were analysed. Changes in the mean Hb level on days 0 and 28 were compared using paired t tests. The relationship between the patient’s baseline parasite load and age was compared using Pearson's correlation. In all analyses, a p value < 0.05 was considered to indicate statistical significance.

### Ethical clearance

Ethical and scientific approval was obtained from the Scientific and Ethical Review Office (SERO) of the Ethiopian Public Health Institute and the Ethics Review Board of the School of Pharmacy and Pharmacology, Addis Ababa University ERB/SOP/409/14/2021. The Arba Minch Zuria health office and the Shecha Health Centre, where the study took place, participants were asked for their permission before the study commenced. All the adult patients provided written informed consent. Assent was obtained from children between the ages of 12 and 17 years. For children under the age of 12, parental or guardian consent was obtained.

## Results

### Enrolment and follow-up

**Fig. 2:**
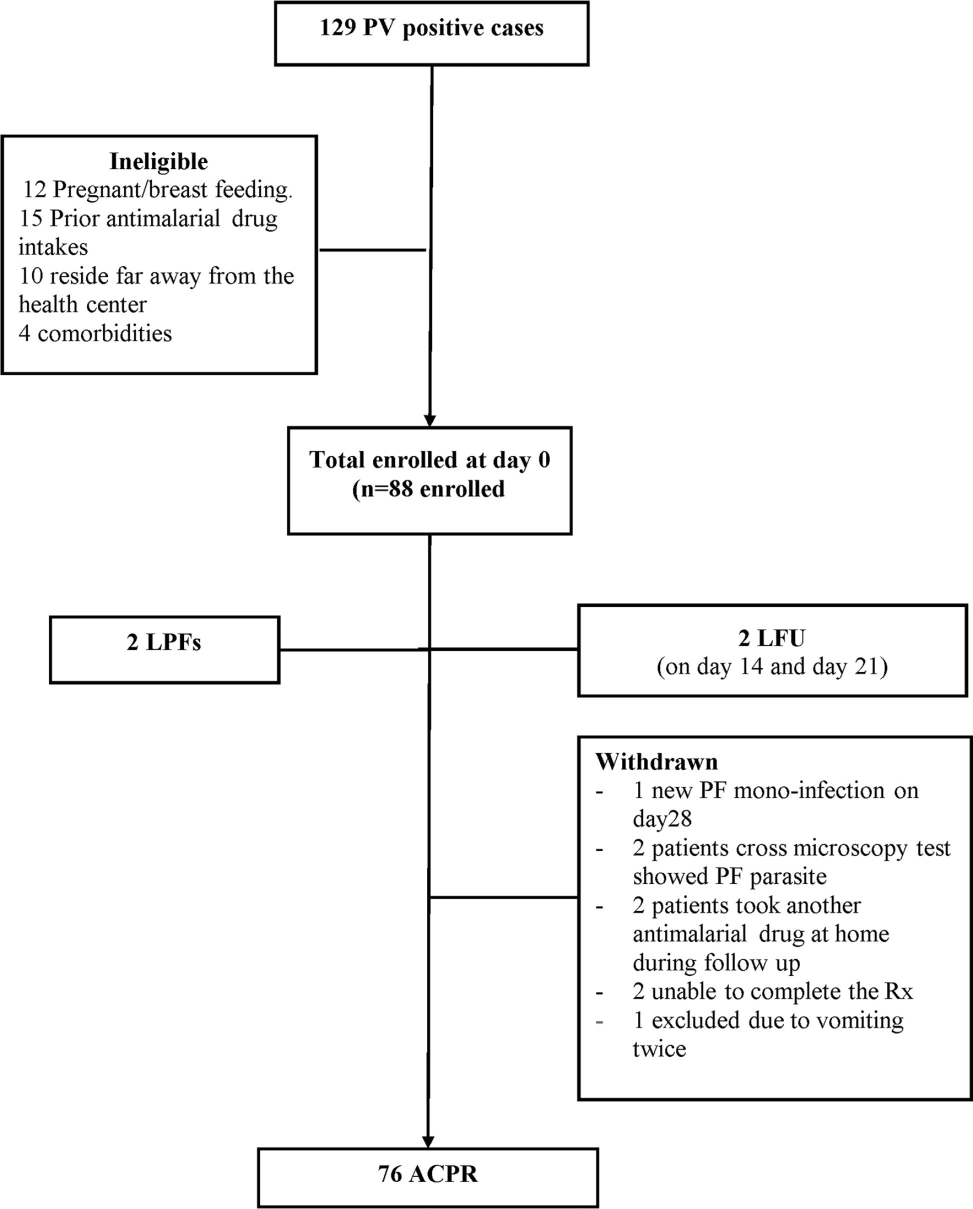
Flow chart of the study participants enrolment and follow up in the study of CQ therapeutic efficacy for treatment of PV malaria cases at Shecha health centre, Arba Minch, Southern Ethiopia. *Rx treatment, PVPlasmodium vivax, PF Plasmodium falciparum, LFU* Lost to follow-up, *LPF* late parasitological failure, *ACPR* adequate clinical and parasitological response

Peripheral blood samples from 3188 patients with suspected malaria were collected and tested for malaria; parasitaemia was detected in 463 (14.5%) patients, 63.5% (294) of whom had *P. vivax* mono-infections, 32.4% (150) had *P. falciparum* mono-infections, and 4.1% (19) had mixed *P. vivax* and *P. falciparum* infections. A total of 88 patients met the criteria and were included in the efficacy study. Ten patients were excluded, eight were withdrawn, and two were lost to follow-up, resulting in 78 patients completing the 28 days of follow-up (Fig. 2).

### Baseline characteristics of the study participants

Of the 88 participants in the study, 62.5% (55/88) were male. The median age of the participants was 16 (range = 1–56), with 56.8% (50) aged 15 years or older, 23.9% (21) aged between 5 and 14 years and 19.3% (17) under 5 years. The mean weight of the study participants was 40.1 kg. The mean axillary body temperature on day 0 was 38.50 °C, and 78.4% (69) of the participants had fever documented at presentation.

**Fig. 3:**
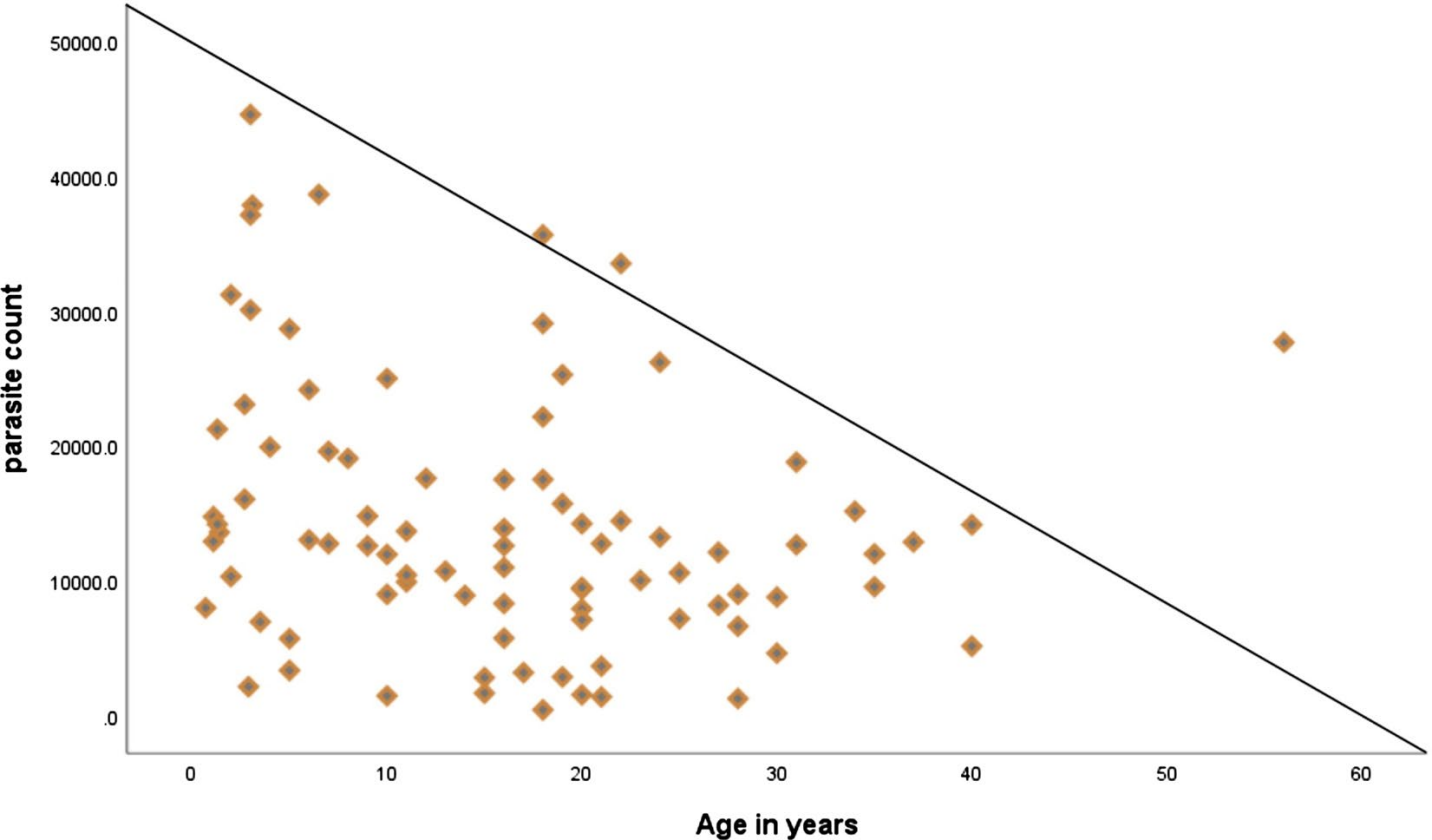
Relationship between parasitaemia and age group at day of enrolment of study participants at Shecha health centre, Arba Minch, southern Ethiopia, 2021

The geometric mean parasitaemia concentration at baseline was 10,691/μl (range = 440–44,564) and varied significantly with age, with the highest value occurring in children < 5 years old (19,089/μl [range = 2,160–44,564]) (p = 0.008) (Table 1 and Fig. 3). The mean Hb concentration at baseline was 12.28 g/dl (Table 1).

**Table 1:** Baseline characteristics of the study participants in the therapeutic efficacy study of CQ for the treatment of uncomplicated *P. falciparum* malaria at Shecha Health Centre, Arba Minch, Ethiopia, 2021

Variables	Age group			
	Under 5	5–14	≥ 15	Total
Gender				
M (%)	10 (58.8)	16 (76.2)	29 (58)	55 (62.5)
F (%)	7 (41.2)	5 (23.8)	21 (42)	33 (37.5)
Median age in years (range)	2.7 (1–4)	9 (5–14)	21 (15–56)	16 (1–56)
Mean Weight in kg (SD)	11.8 (2.7)	26.2 (8.4)	55.6 (7.5)	40.1 (19.8)
Mean Temperature in ^0^C (SD)	38.7 (1.1)	38.7 (1.2)	38.4 (1.1)	38.5 (1.1)
Geometric mean of Parasitaemia (per μl),	16,337.8	12,120	8779.7	10,691
Gametocyte carriage (per μl) (range)	7215 (2160–42699)	4737 (0–25200)	1364 (0–6280)	3299.3 (0–42699)
Mean Hemoglobin in g/dl (SD)	11 (0.7)	11.5 (1.5)	13 (1.2)	12.28 (1.5)

*M* male, *F* female, *kg *kilogram, *SD* standard deviation, *°C* degree centigrade, *g/dl* gram/decilitre, *μl* microlitre

During the CQ treatment (day 0, 1, and 2), 15.9% (14/88) of the patients vomited their dose of chloroquine at least once, of whom 64% (9/14) vomited on day one. Retreatment was prescribed for four of the 14 patients who vomited within 30 min. One of the patients vomited twice on day 0, just after he took CQ tablets, and he was withdrawn from the study.

### Treatment outcomes

#### Efficacy end points

**Fig. 4:**
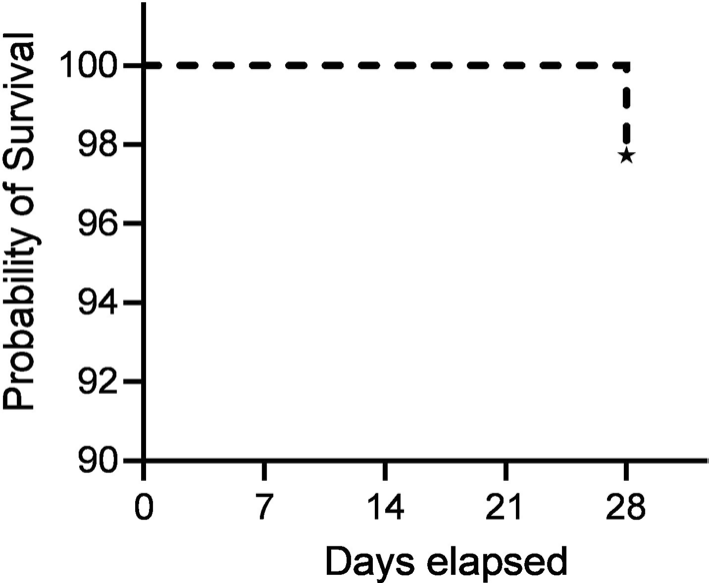
Kaplan–Meier survival curve

Of the 78 patients who completed treatment and subsequent follow-up, two (2.6%) had *P. vivax* parasitaemia during follow-up without clinical symptoms (LPF); both recurrences occurred on day 28, both were under 5 years of age, and they took a 24.1 mg/kg total dose (Table 2). There were no cases of early treatment failure (ETF) or late clinical failure (LCF). Eighty-eight patients were included in the survival analysis. On day 28, the cumulative risk of recurrence following CQ treatment was 2.5% (95% CI, 0.6–9.5%) (Fig. 4).

**Table 2:** The 28-day CQ therapeutic efficacy outcome in uncomplicated *P. vivax* malaria patients at the Shecha Health Centre, Arba Minch, Ethiopia, 2021

	N	%	Lower 95% CI	Upper 95% CI
ETF	0	0.0		
LCF	0	0.0		
LPF	2	2.6	0.3	9.0
ACPR	76	97.4	91.0	99.7
WTH	8			
LFU	2			
Total WTH & LFU	10	11.4		
Total patients at baseline	88			
Total patients per protocol	78			

*ETF* Early treatment failure, *LCF* Late clinical failure, *LPF* Late parasitological failure, *ACPR* Adequate clinical and parasitological response, *LFU* Loss to follow-up, *WTH* withdrawal

#### Parasite and fever clearance

On the day of enrolment, participants had a mean parasitaemia of 16,337.8/μl (range 440–44564). The incidence of parasitaemia on the day of enrolment was significantly greater in the < 5 years of age group than in the other age groups (p = 0.008) (Table 1 and Fig. 3). All patients had cleared parasitaemia by day two. Except for two LPF patients, no other patients showed parasitaemia after day two (Tables 2, 3).

**Table 3:** CQ treatment outcome by age category in *P. vivax*-infected patients at the Shecha Health Centre Arba Minch, southern Ethiopia (2021)

Outcome	Age category in years			
	< 5	5–14	≥ 15	Total
	n (%)	n (%)	n (%)	n (%)
ACPR	13 (16.7)	19 (24.3)	44 (56.4)	76 (97.4)
LPF	2 (2.6)	0	0	2 (2.6)
WTH	2	2	4	8
LFU	0	0	2	2

In total, 78.4% (69/88) of patients had documented fever at baseline, with a mean temperature of 38.5 °C. The other patients had a fever history 24 h prior to enrolment. Among those presenting with fever, 73.9% (51/69), 97.1% (67/69) and 100% were afebrile on days 1, 2 and 3, respectively. After day two, no fever was recorded in any patient.

#### Gametocyte carriage

**Fig. 5:**
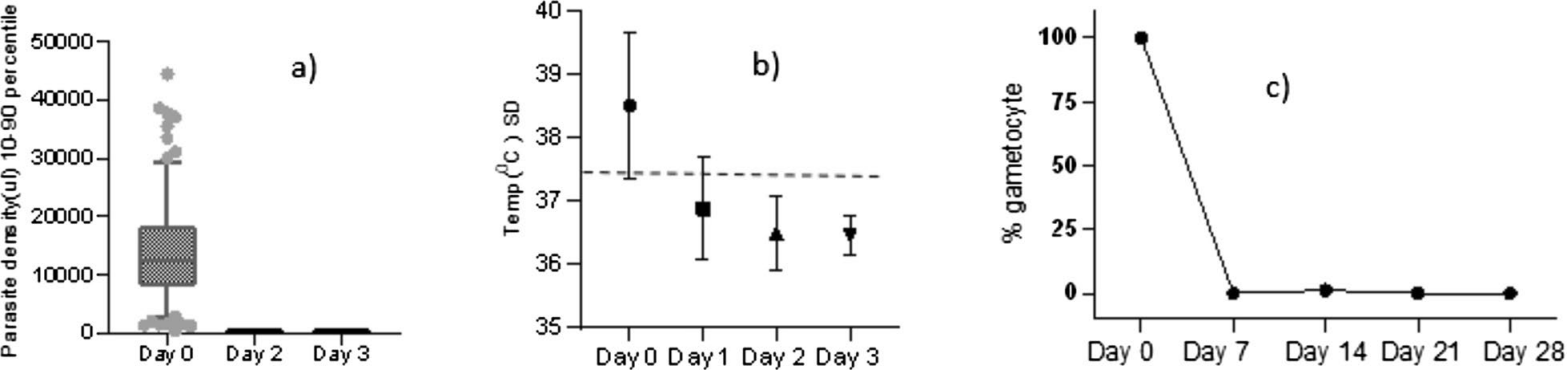
Pattern of parasite (**a**) fever (**b**), and gametocyte (**c**) clearance in *P. vivax* infected patients treated with chloroquine in Arba Minch, Southern Ethiopia, 2021

Gametocytes were detected in 95.5% (84/88) of the participants at enrolment, with a mean gametocytaemia of 3,299.3/μl (range = 0–42,699). Gametocyte clearance was rapid, and by day two, only 3 study participants had patent peripheral gametocytaemia. All the gametocytes were cleared on the third day of follow-up, and no new gametocyte carriers were observed thereafter (Fig. 5).

#### Haemoglobin recovery

**Fig. 6:**
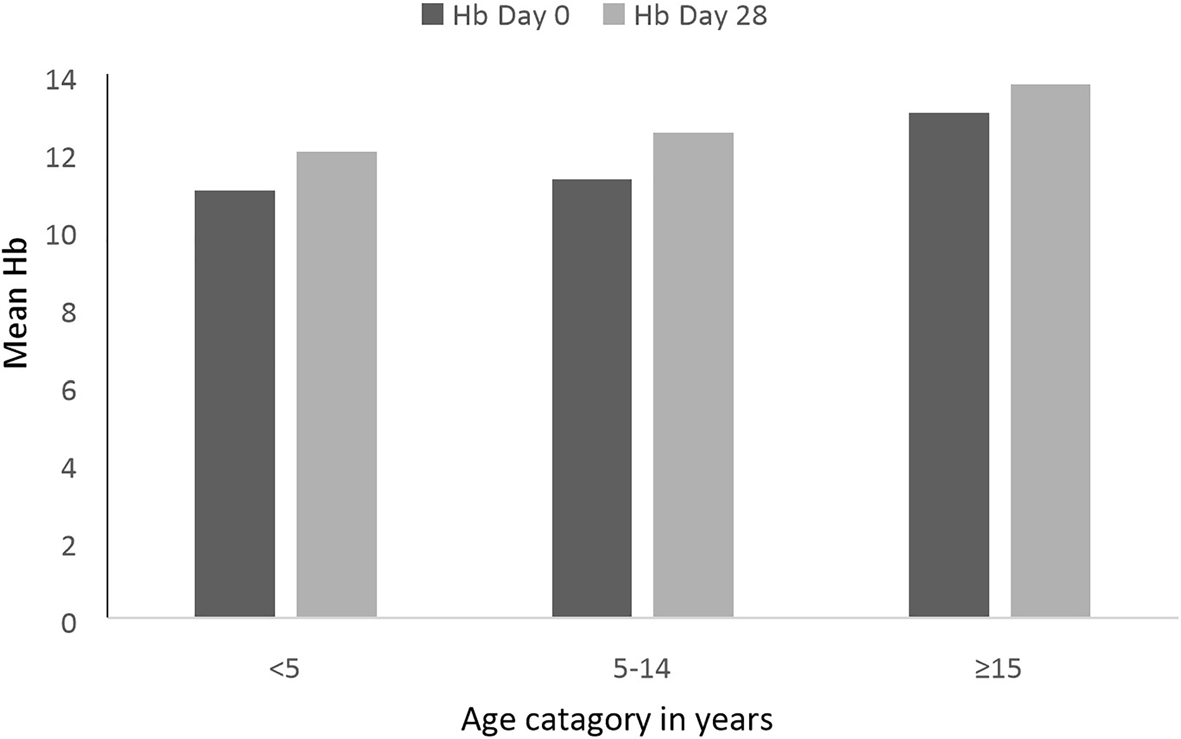
Haemoglobin recoveries among study participants in different age groups at Shecha health centre, Arba Minch, Southern Ethiopia, 2021. *Hb* haemoglobin

The mean Hb concentration at enrolment was 12.28 g/dl (range: 8.30–15.00 g/dl) and increased to 12.53 g/dl (9.6–15.3 g/dl) on day 14 and 12.9 g/dl (10–16 g/dl) on day 28 (p = 0.001). There was no difference between the Hb levels on day 0 and on the day of treatment failure (day 28) for the two patients whose treatments failed. The Hb concentration in children under 5 years old rose from 11.0 on day 0 to 12.1 g/dl on day 28. The corresponding increase in patients aged 5–14 years was 11.5 to 12.6 g/dl, and that in patients aged 15 years and above was 13 to 13.5 g/dl. Hb recovery was significant (p < 0.001) (Fig. 6).

### Adverse events

A total of 49 adverse events attributed to CQ were recorded in 32 (36.4%) of the 88 patients. Among the adverse events, vomiting (17%) and headache (10.2%) occurred most frequently. Coughing (5.7%) and dizziness (5.7%) were also reported. Most of the adverse events (87.8%, 43/49) were reported during the first week of follow-up (Table 4). All of the itching (4.5%) and diarrhoea (1.1%) patients were treated with rescue treatment (cetirizine for itching and ciprofloxacin for diarrhoea), but the other reported adverse events resolved without any treatment.

**Table 4:** Frequency of adverse events during the 28-day follow-up of CQ at Shecha Health Centre, Arba Minch, southern Ethiopia

Adverse event	Days of the follow up and frequency of adverse events								Total n (%)
	Day 0	Day 1	Day 2	Day 3	Day 7	Day 14	Day 21	Day 28	
Headache		5 (5.7)	2 (2.3)		1 (1.1)		1 (1.1)		9 (10.2)
Anorexia			1 (1.1)						1 (1.1)
Vomiting	1 (1.1)	9 (10.2)	4 (4.5)	1 (1.1)					15 (17)
Abdominal pain				1 (1.1)					1 (1.1)
Diarrhoea				1 (1.1)					1 (1.1)
Cough					1 (1.1)	2 (2.3)	2 (2.3)		5 (5.7)
Dizziness		1 (1.1)		1 (1.1)	3 (3.4)				5 (5.7)
Myalgia				1 (1.1)					1 (1.1)
Itching		1 (1.1)	3 (3.4)						4 (4.5)
Behavioural change		1 (1.1)							1 (1.1)
Bitter taste			1 (1.1)						1 (1.1)
Fever			1 (1.1)		1 (1.1)	1 (1.1)			3 (3.4)
Rash			2 (2.3)						2 (2.3)
Total n (%)	1 (1.1)	17 (19.3)	14 (15.9)	5 (5.7)	6 (6.8)	3 (3.4)	3 (3.4)		49 (55.7)

## Discussion

Malaria caused by *P. vivax* is endemic throughout the Horn of Africa, primarily in Ethiopia, where CQ remains the mainstay for blood-stage treatment [1]. With increasing reports of CQ resistance in vivax-endemic sites across the globe, the threat of CQ failure is a major concern in Ethiopia. This therapeutic efficacy survey revealed that CQ remained effective for the treatment of *P. vivax* malaria in southern Ethiopia in 2021.

Several *P. vivax* CQ efficacy surveys have been conducted in Ethiopia in the past decade [17, 18, 21–25]. Most of these studies have reported a treatment failure rate between 0 and 4.6%, which is in line with the current finding. On the other hand, some studies have reported a high failure rate, ranging between 6 and 21.9% [19, 20, 31]. Notably, Getachew et al. studied in four sentinel sites and found different results, the treatment failure rate was 3.8% in Shele, 5.9% in Batu, 9.2% in Shone and 21.9% in Guba, but the overall study’s failure rate was 9.4% [19]. In the present study, of the 78 study participants who completed the study, 97.4% (95% CI, 91.0–99.7%) had ACPR, and 2.6% had treatment failures. Both treatment failures occurred on day 28 without any malarial symptoms and were classified as LPF. These two treatment failures were observed in patients younger than 5 years of age, in line with the findings of other studies [32, 33]. The possible causes for the two treatment failures could be malabsorption, recrudescence, relapse, or reinfection. Given the lack of drug level data available and the difficulty in genotyping relapse infection in *P. vivax*, the apparent cause of LPFs could not be identified.

Overall, CQ appeared to be highly effective at treating *P. vivax* infections in the study area. The cure rate in the present study was 97.4%, which is in accordance with the findings of a similar recent study in Shewa Robit, central Ethiopia, in which 98.8% efficacy was reported [24]. Despite the few reports of severe treatment failure (above 10%), most studies conducted in Ethiopia have reported low treatment failure; thus, CQ remains the treatment of choice for *P. vivax* malaria. When CQ is effective, this policy has the advantage of lower cost relative to ACT, and its long half-life has been shown to afford a greater reduction in *P. vivax* recurrence relative to ACT when combined with PQ for radical cure [34]. However, with reports of high-grade CQ resistance in other regions of the globe and the risk of resistance spreading or local emergence, ongoing surveillance in Ethiopia is warranted. Several countries have reported very high failure rates—10.1% in Brazil [35]; 17.5% in Timor-Leste [36]; 34% in Myanmar [37]; 61% in eastern Malaysia [15]; and 84% in Papua Indonesia [38]. In Indonesia, Papua New Guinea and Malaysia, high-grade treatment failure (exceeding the WHO guideline by more than 10% of national treatment failure) resulted in an anti-malarial policy change to a universal ACT policy for all malaria species [1, 29].

The geometric mean parasitaemia concentration on the day of enrolment was 10,691/μl, which is comparable with that reported in other studies performed in Ethiopia, with a study in Shewa Robit reporting 8723.9/µl [31] and one in Illubabore reporting 11,316 per microlitre [25]. However, other studies reported lower geometric mean parasite density, which ranged between 2270 and 6614 [17, 18, 21, 24, 39]. The current study showed that parasite density at baseline was greater in children < 5 years of age than in individuals in other age categories. This finding is similar to those of other studies in Ethiopia, including a study in Benishangul Gumuz, Ethiopia [39]. However, a 2017 study by Shewa Robit reported that there was no correlation between age and parasite load [31].

In the present study, fever, parasitaemia and gametocyte clearance were achieved within 72 h of CQ treatment. The clearance rate is similar to that reported in a few other studies performed in Ethiopia [23, 31]. However, some studies reported that the fever and parasites were completely cleared on day 7 [17, 18, 22, 25].

A significant Hb recovery was observed in this study, with a mean Hb level of 12.28 g/dl on day 0 increasing to 12.9 on day 28. Similar results were reported by other studies, including those of Shumbej et al*.* [21], who reported an increase from 11.8 to 13.8 g/dl; Assefa et al*.,* who observed an increase from 11.5 to 13.4 g/dl [23]; and Beyene et al*.,* who observed an increase from 12.2 to 13.3 g/dl [39]. However, other studies have not reported significant Hb recovery from day 0 to day 28 [16, 22]. Hb did not improve in two patients with treatment failure; in one of the patients, the amount of Hb decreased, while it remained unchanged in the other patient.

In this study, malaria was common among people presenting with acute febrile illness at Shecha Health Centre during the study period, accounting for 14.5% of febrile illnesses. Of these, 63.5% (294/463) had *P. vivax* mono-infections, 32.4% (150/463) had *P. falciparum* mono-infections, and 4.1% (19/463) had mixed (*P. vivax* and *P. falciparum*) infections. These high *P. vivax* prevalence findings are similar to those of other studies conducted in Arsi [40], where 70.4% of infections were *P. vivax* mono-infections, 23% were *P. falciparum* mono-infections, and 6.5% were mixed infections. Similarly, in Halaba [41], 74% of infections were *P. vivax* mono-infections, 19.8% were *P. falciparum* mono-infections, and 6.2% were mixed infections. The *P. vivax* prevalence is much greater than that reported in other parts of Ethiopia, where *P. falciparum* is the dominant *Plasmodium* species (> 60%) [3, 42]. This could be because of the season of the study, which ranged from March to August 2021.

In this study, 36.4% of the subjects reported common adverse events associated with CQ. The most frequent adverse event was vomiting. Most of the adverse events were similar to the symptoms of malaria and most resolved in line with malaria recovery and without any rescue treatment. Similar results were reported by Belay et al*.* [24]. However, during the 28-day follow-up, no serious adverse events were reported by the study subjects.

This study has some limitations. Despite several efforts, the blood concentration of CQ was not measured due to certain limitations. Another limitation of this study was that no genotyping was conducted in the two LPF patients to differentiate recrudescence from new infection. New molecular approaches have been established to support the distinction of recrudescence from relapse and reinfection in *P. vivax* recurrences but require genotyping large numbers of markers [43].

## Conclusion

The study showed that the therapeutic efficacy of CQ for the treatment of uncomplicated *P. vivax* malaria mono-infections is high in southern Ethiopia. Following CQ therapy, the clinical, parasitological, and other laboratory parameters improved significantly. Therefore, this study supports the national malaria treatment guidelines of Ethiopia, where CQ is used for the management of *P. vivax* infection. However, given the conflicting results of therapeutic efficacy studies in Ethiopia, nationwide regular surveillance of CQ efficacy supported by advanced molecular and analytic methods is recommended.

## Data Availability

All data presented in this study are contained within the manuscript.
